# Chronic Nonbacterial Osteomyelitis in Inflammatory Bowel Disease

**DOI:** 10.3390/life13122347

**Published:** 2023-12-15

**Authors:** Ariadni Tzaneti, Elli Athanasopoulou, Smaragdi Fessatou, Lampros Fotis

**Affiliations:** 1 Department of Pediatrics, Attikon General Hospital, National and Kapodistrian University of Athens, 124 62 Athens, Greece; 2 Division of Pediatric Gastroenterology, Department of Pediatrics, Attikon General Hospital, National and Kapodistrian University of Athens, 124 62 Athens, Greece; sfessatou@med.uoa.gr; 3 Division of Pediatric Rheumatology, Department of Pediatrics, Attikon General Hospital, National and Kapodistrian University of Athens, 124 62 Athens, Greece

**Keywords:** CRMO, Crohn’s disease, inflammatory bowel disease, osteomyelitis, pediatrics, ulcerative colitis

## Abstract

Chronic nonbacterial osteomyelitis (CNO), also known as chronic recurrent multifocal osteomyelitis (CRMO), is a rare autoinflammatory bone disease primarily affecting children and adolescents. This review presents a comprehensive analysis of the intricate relationship between CNO and inflammatory bowel disease (IBD), shedding light on shared pathophysiological mechanisms and clinical management. A thorough literature review was conducted, encompassing 24 case reports involving 40 patients. The demographic distribution of patients revealed a near-equal gender ratio, with a median age of diagnosis at 12 years. The diagnosis patterns showed a higher proportion of CNO as the initial diagnosis, while Crohn’s disease was more prevalent than ulcerative colitis. The time interval between the clinical presentations varied, ranging from simultaneous detection to a substantial 15-year gap. Treatment modalities included nonsteroidal anti-inflammatory drugs (NSAIDs), steroids, aminosalicylates, and biologic agents, such as infliximab, often overlapping in their use and suggesting shared pathophysiological pathways. Both conditions displayed systemic manifestations, and patients often responded well to immunosuppressive medications. The pathophysiology of CNO involves a genetic predisposition, cytokine dysregulation, and osteoclast activation. Dysregulated innate immunity results in immune cell infiltration into bones, causing sterile bone lesions. Notably, emerging evidence hints at a potential link between the microbiome and CNO. In contrast, IBD results from imbalanced mucosal immune responses to the intestinal microbiota. Polymorphisms in the promotor region of IL-10, common cytokines, immune cells, and genetic markers indicate shared immunological and genetic factors between CNO and IBD. Both conditions also involve extraintestinal symptoms. This analysis underscores the need for clinical awareness of the co-occurrence of CNO and IBD, especially among pediatric patients. A deepened understanding of the connections between these seemingly distinct diseases could lead to more effective management and improved patient outcomes.

## 1. Introduction

Chronic nonbacterial osteomyelitis (CNO), also known as chronic recurrent multifocal osteomyelitis (CRMO), is a rare autoinflammatory bone disease that primarily affects children and adolescents. Despite its rarity, CNO can significantly impact the lives of young patients and their families [1]. Inflammatory bowel disease (IBD), a group of chronic inflammatory disorders affecting the gastrointestinal tract, is a condition that shares intriguing similarities with CNO. Notably, while IBD primarily involves the gut and CNO affects the bones, there is a growing body of evidence pointing to overlapping pathophysiological mechanisms between these seemingly distinct conditions. Commonalities observed in genetics, environmental influences, microbiome dysbiosis, systemic manifestations, and responses to treatment suggest potential shared pathophysiological mechanisms [1]. The primary objective of this analysis is to consolidate and elucidate the information surrounding these two medical conditions by shedding light on any discernible patterns, connections, or insights revealed by prior research and to contribute to the existing body of knowledge in the medical field, fostering a deeper understanding of the relationship between CNO and IBD.

## 2. Methods

We conducted a search of the Medline-indexed literature on PubMed by combining the following search terms and keywords: “Chronic Recurrent Multifocal Osteomyelitis”, “Chronic Nonbacterial Osteomyelitis”, “CNO”, “CRMO”, “CRMO and IBD”, “CNO and IBD”, “Inflammatory Bowel Disease and Osteomyelitis”, “Crohn’s Disease and CRMO”, “Ulcerative Colitis and CRMO”, “Crohn’s Disease and CNO”, and “Ulcerative Colitis and CNO”. We also conducted a supplementary search by examining the reference lists of identified articles for additional papers. Our inclusion criteria encompassed case reports or case series, cohort studies, and clinical studies that investigate the co-occurrence or association between CNO and IBD, as well as articles providing detailed clinical and diagnostic information on patients with both CNO and IBD. We limited our results to English language publications. Exclusion criteria were applied to studies not directly related to the association between CNO and IBD, articles lacking sufficient clinical information, and duplicates or those with overlapping data. Two independent reviewers performed the initial screen of articles based on their titles and abstracts. Full-text articles were subsequently reviewed to determine their eligibility for inclusion in this literature review. Any differences in judgment were resolved through discussion and consensus.

## 3. Results

A comprehensive review of 24 case reports involving a total of 40 patients shed light on the intricate correlation between IBD and CNO [2,3,4,5,6,7,8,9,10,11,12,13,14,15,16,17,18,19,20,21,22,23,24,25] (Table 1 and Table 2). Among these patients, 22 (55%) were females and 18 (45%) were males, reflecting a nearly equal gender distribution. The median age of the patients was 12 years, with ages ranging from as young as 9 months to as old as 41 years. The analysis revealed distinct patterns in the diagnosis of CNO and IBD among the study population. A significant majority of patients, numbering 21 (52.5%), received their initial diagnosis of CNO. Conversely, in 14 patients (35%), the diagnosis of IBD proceed the one of CNO. A smaller subset of 5 patients (12.5%) received concurrent diagnoses of both chronic CNO and IBD. Further delineating the IBD cases, 23 patients (57.5%) were diagnosed with Crohn’s disease, while 15 patients (37.5%) presented with ulcerative colitis and 2 of them (5%) were identified as unclassified IBD (Table 3). This conclusion aligns with the percentages attributed in the review by Costi et al. (2023) [26]. The median time interval between the two clinical presentations was 2 years, encompassing a spectrum ranging from concomitant diagnosis to an extended 15-year duration. Among the 40 reported cases of chronic nonbacterial osteomyelitis (CNO), the diagnostic breakdown based on radiological methods was as follows: X-rays in 7 cases (17.5%), bone biopsy in 22 cases (55%), magnetic resonance imaging (MRI) in 32 cases (80%), and PET-CT scan in 1 case (2.5%). The distribution of affected bones appeared to be diverse, although certain bones were affected more frequently than others. Among the reported cases, the distal and proximal femur were involved in 14 out of 40 cases (35%), the tibia in 12/40 (30%), the fibula in 4/40 (10%), the sacroiliac joint in 4/40 (10%), and the clavicles in 4/40 (10%). These bones were consistently mentioned as involved in the reviewed cases. The bilateral nature of involvement in specific areas, such as the distal femoral metaphysis and proximal tibial metaphysis, indicates a pattern. However, further statistical analysis would be needed for a more precise assessment of the frequency of bone involvement in this specific context.

Clinical interventions administered for the management of these conditions included various therapeutic options. For CNO, nonsteroidal anti-inflammatory drugs (NSAIDs) were administered to 17 patients, representing 42.5% of cases. A biphosphonate such as zoledronic acid was prescribed to a single patient and pamidronate acid was employed in 4 cases, addressing CNO in 19.0% of instances. For IBD, corticosteroids were employed in the management of 24 patients with both CNO and IBD (60%). Aminosalicylates were prescribed to 18 patients (45%) to address both CNO and IBD concurrently. Infliximab was used in 10 cases (25%) as a treatment for IBD or/and CNO, and adalimumab was utilized in 5 cases. Additionally, tumor necrosis factor α inhibitor (anti-TNF-α) therapy was administered to two more patients without specifying the specific agent utilized. Azathioprine was also administered to 11 patients (27.5%) for the treatment of IBD. Finally, methotrexate was employed in 10 patients (25%) for the management of both CNO and IBD. In two instances (5%), colectomy was performed as a necessary surgical intervention due to the severity of IBD [5,10]. Additionally, one patient within the cohort was identified as having Takayasu arteritis and received intravenous immunoglobulin (IVIG) therapy, underscoring the complexity of overlapping autoimmune conditions [16]. Different conditions coexisted in several cases, with primary sclerosing cholangitis being the most common in 3 out of 40 cases each [4,8,21], and others such as psoriasis (2/40) pyoderma gangrenosum (1/40) [3], Takayasu arteritis (1/40) [16], and eosinophilic esophagitis (1/40) [11] (Table 4). Cantarelli et al. describe the successful treatment of refractory Crohn’s disease in combination with CNO in a 10-year-old patient using a combination therapy of infliximab, methotrexate, and Crohn’s disease exclusion diet plus partial enteral nutrition, as suggested by Levine et al. [22,27]. It is important to note that in eight case reports (20%, 8/40), intravenous antibiotics were reported to be used for the treatment of osteomyelitis before arriving at the diagnosis of CNO. In terms of laboratory findings, there were no specific autoantibodies noted to be elevated in most cases of concurrent CNO with IBD, except for positive ANCA antibodies identified in four case reports [3,4,8,11].

## 4. Discussion

Kahn et al. [28] first described a concurrent diagnosis of CNO and IBD. Following that, an expanding body of literature has emerged, including case reports and case series detailing this connection. While most of these reports center on pediatric cases, it is noteworthy that in reported instances, the initial symptoms manifested after individuals reached 18 years of age. CNO particularly affects children, but it can persist in adulthood or present later in life. The average age at diagnosis is 9–11 years [29], but the delay in diagnosis from the onset of symptoms is usually around 1 year [30,31]. The incidence of the disease is estimated to be 0.4 per 100,000 children [30,32] with a slight predominance of females than males [33]. However, in series from India and Japan, a male predominance is observed [34]. The actual prevalence of CNO is probably understated in earlier studies due to advancements in imaging and diagnostic methods, as well as the notable surge in noninfectious osteomyelitis case reports and series during the past decade [31]. It can affect all ethnicities, although the White Caucasian population is most frequently affected [35].

While the reported incidence of chronic nonbacterial osteomyelitis (CNO) has been as low as four per million children, there is a noticeable rise in cases across various centers, coinciding with increased awareness [32]. The exact prevalence of coexisting IBD in children with a CNO diagnosis is not well documented. Conversely, among children with known IBD, there are patients where both diagnoses coexist. The exact prevalence has also not been documented, as the coexistence of the two diseases has recently been established. Furthermore, in certain cases, symptoms of CNO may be recorded as extraintestinal manifestations of IBD, which underestimates the coexistence rates of the two clinical entities. A retrospective chart review of patients with a diagnosis of either IBD or CRMO was performed in 2021. Among 600 pediatric patients diagnosed with IBD and 47 with CRMO, seven pediatric cases revealed a concurrent diagnosis of both IBD and CRMO. The identified percentage of pediatric patients with IBD that also had CRMO was 1% [23]. Within the existing literature, there is an absence of data regarding the percentage of IBD occurrence in patients initially diagnosed with CNO. No information has been published or made available to date, leaving a gap in our understanding of this specific aspect of the condition. Further research is needed to fill this void and provide a more comprehensive insight into the prevalence of IBD in patients diagnosed with CNO.

In this comprehensive analysis of 24 case reports, the complex relationship between IBD and CNO, especially in pediatric patients, is emphasized. The demographic distribution reveals a nearly equal gender ratio, with a median age at diagnosis of 12 years. The diagnostic sequence shows a higher proportion of CNO as the initial diagnosis, while Crohn’s disease was more prevalent than ulcerative colitis. Additionally, the temporal pattern of diagnosis ranged from simultaneous detection to a substantial time gap of 15 years between the clinical presentation of symptoms. The therapeutic approach encompassed a range of medications, with steroids being the most commonly used for both CNO and IBD cases. NSAIDs, steroids, aminosalicylates, and biologic agents (with infliximab being one of the most prominent) are the most used drugs in the treatment of both conditions. They often overlap in their therapeutic uses, suggesting that they may follow a similar pathophysiological or molecular pathway.

The clinical presentation of CNO can vary widely from one patient to another, making it a challenging diagnosis to establish. Common symptoms include focal bone pain and tenderness at the affected sites [29,36]. However, further musculoskeletal symptoms, such as swelling, skeletal deformities, and joint pain, may also be involved. The most commonly affected sites are long bones, particularly the epiphyses and metaphyses, with the femur, tibia, and pelvis being the most frequently involved [1,37]. These symptoms can be intermittent and may occur in flares. CNO can affect multiple bones simultaneously, a phenomenon known as multifocality, or it may involve a single bone. Some patients may also experience multifocal lesions that are gradually diagnosed during their follow-up [38]. Systemic symptoms, such as fever and fatigue, are less frequently observed and may raise a suspicion of alternative diagnoses [1,39]. Cutaneous manifestations may also be present, including acne, psoriasis, palmoplantar pustulosis, and pyoderma [1]. Skin involvement is further encountered in the context of the Synovitis, Acne, Pustulosis, Hyperostosis, Osteitis (SAPHO) symptom complex, which could be considered the adult form of CNO [40].

Clinical features in individuals with CNO alone may predominantly revolve around bone-related symptoms, such as localized pain, swelling, and tenderness [41]. On the other hand, in cases of IBD alone, clinical features may involve gastrointestinal symptoms like abdominal pain, diarrhea, weight loss, and fatigue. Extraintestinal manifestations, including joint pain and inflammation, can also occur and overlap with CNO manifestations [42]. Individuals with both CNO and IBD may exhibit a unique set of clinical features that overlap or manifest differently compared to cases of CNO or IBD alone. These features could include bone pain, gastrointestinal symptoms, and signs of systemic inflammation [26].

The distribution and distribution frequency of lesions in patients experiencing both CNO and IBD co-occurrence closely mirrors those observed in individuals diagnosed solely with CNO [41]. The similarity in the lesion distribution suggests a potential association between the two conditions. However, further comprehensive studies and analyses are essential to elucidate any distinct patterns or variations in lesion distributions within the context of concurrent CNO and IBD, contributing to a deeper understanding of their interconnected pathophysiology.

Several factors are known to be involved in the pathophysiology of CNO. Dysregulated innate immunity results in immune cell infiltration into the bones, subsequently activating osteoclasts and causing sterile bone lesions [12]. One central aspect of the condition is the disrupted balance of cytokines and chemokines within the immune cells. In patients with CNO, immune cells called monocytes show reduced levels of immune-regulating cytokines like IL-10 and IL-19, while displaying elevated levels of pro-inflammatory cytokines (IL-1β, IL-6, TNF-α) and chemokines (IL-8, IP-10, MCP-1, MIP-1a, MIP-1b). This imbalance is partially due to a faulty activation of specific signaling pathways, leading to reduced expression of regulatory factors (Sp-1) that control the production of IL-10 and IL-19. Additionally, this impaired signaling affects histone proteins, which regulate gene expression. This chain reaction results in altered gene expression patterns, further complicating the immune response [1,6].

Another crucial observation is the increased activity of the NLRP3 inflammasome, a complex that triggers the release of the highly inflammatory IL-1β. This molecular imbalance does not just impact the immune system but also affects osteoclasts, cells responsible for bone maintenance. While some individuals with CNO have a clear genetic component or are linked to other autoimmune or inflammatory disorders, many cases do not stem from a single genetic mutation. These cases involve a combination of genetic predisposition and environmental factors, making it challenging to pinpoint specific genes responsible for the condition [43]. However, certain syndromic forms of CNO, like Majeed syndrome and deficiency of the IL-1 receptor antagonist (DIRA), have been linked to specific gene mutations (LPIN2 and IL1RN), shedding light on the role of IL-1β in inflammation. It is important to note that these mutations explain only a minority of CRMO cases, and for most patients, the genetic factors predisposing them to the disease remain unclear [44].

Zhao et al. [43] propose a potential link between the microbiome and CNO. Emerging evidence suggests that interactions with these microorganisms may impact the immune balance and potentially play a role in disease onset. Animal model research has demonstrated that manipulating the microbiome can prevent osteomyelitis. The gut microbiome is known to play a crucial role in immune system modulation, and alterations in the composition and function of the gut microbiota have been implicated in IBD [45]. It is conceivable that disruptions in the balance of gut microorganisms could extend beyond the intestinal environment and contribute to the development of bone inflammation in CNO. This theory suggests a potential systemic effect where changes in the gut microbiota influence immune responses in distant tissues. In patients with CNO, a compromised intestinal barrier could lead to inflammation, resulting in the release of cytokines. These cytokines may play a role in triggering extraintestinal manifestations of inflammatory bowel disease (IBD), with a specific emphasis on bone inflammation [26].

Eventually, the pathophysiology of CNO involves a complex interplay of genetic predisposition, cytokine dysregulation, osteoclast activation, and potentially, interactions with the microbiome. While much progress has been made, further research is needed to fully understand the intricate mechanisms underlying this enigmatic condition. While the molecular pathophysiology remains partially understood, it is intriguingly linked to other auto-inflammatory conditions like inflammatory bowel disease (IBD), psoriasis, Wegener’s disease, and SAPHO syndrome. Chronic nonbacterial osteomyelitis (CNO) may manifest several years before the symptoms of the associated disease, and the bone remodeling induced by CNO can lead to lasting disability [46,47].

While the precise mechanisms linking bone inflammation in chronic nonbacterial osteomyelitis (CNO) and intestinal inflammation in inflammatory bowel disease (IBD) are not fully elucidated, several theories have been proposed to shed light on their potential connections. Inflammatory bowel disease (IBD) is thought to result from an imbalanced response of the mucosal immune system to the intestinal microbiota. This dysregulation can manifest as both excessive immune reactivity and inadequate immune responses. It is suggested that both CNO and IBD may stem from an abnormal immune response, leading to inflammation in distant sites, such as bones and the intestine. This shared dysregulation could involve the release of pro-inflammatory cytokines and other immune mediators that contribute to the inflammatory processes observed in both conditions [48]. Dysregulation of IL-10 is a shared pathophysiological mechanism between CNO and IBD, as polymorphisms in the promotor region of IL-10 have been observed in IBD patients [49]. Additionally, the link between IBD and musculoskeletal manifestations, is well established, indicating a gut-synovial axis [50], while some reports even suggest CNO as an extraintestinal IBD manifestation [17]. Gut immune responses can affect the joints, with TNF-α, IL-12 and IL-23 playing crucial roles. TNF-α upregulation is present in IBD, arthritis and CNO, explaining the positive response to anti-TNF-α [23]. IL-12 has been noted as a marker for the treatment response in CNO patients [51]. IL-23 has not been measured in patients with CNO; however, ustekinumab, an IL-12/IL-23 inhibitor, showed promise in one patient, prompting further study [23]. Common cytokines and immune cells are implicated in both conditions, as mentioned above, suggesting a shared immunological basis. Moreover, there is evidence of a genetic predisposition to both CNO and IBD. Certain genetic markers and susceptibility genes have been identified for both conditions, indicating potential shared genetic factors. The NLRP3 gene mutations associated with autoinflammatory syndromes have been found in some CNO patients, as mentioned, and similar genetic mutations are implicated in IBD [1]. In relation to genetic factors, a limited group of patients with CNO underwent testing for well-known susceptibility genes associated with IBD, such as the CARD15/NOD2 gene [9]. Nevertheless, no significant association was detected. Further research is needed to unravel the intricate connections between these two seemingly distinct inflammatory disorders. Both CNO and IBD can have systemic manifestations beyond their primary sites of inflammation. For example, patients with IBD may experience extraintestinal symptoms like joint inflammation (arthritis), skin conditions (psoriasis), and eye inflammation (uveitis). Similarly, CNO can lead to musculoskeletal symptoms beyond bone inflammation [28,46,47]. Furthermore, patients presenting with both CNO and IBD often respond positively to immunosuppressive medications that help control inflammation. Medications such as corticosteroids, immunomodulators, and biologic factors are commonly used in the treatment of both conditions [46,52].

CNO should be considered as a potential diagnosis in a pediatric patient experiencing recurring or constant localized discomfort in the bones or joints of the lower limbs, clavicle, spine, or jaw. Some patients might also experience localized swelling and increased warmth in the affected region. This pain tends to intensify at night, potentially leading to sleep disruption [26]. A laboratory evaluation is necessary to complement the diagnostic process. A complete blood count (CBC), C-reactive protein (CRP), and erythrocyte sedimentation rate (ESR) should be routinely performed to assess the possibility of infectious osteomyelitis. It is worth noting that most children with CNO typically present with normal CBC, CRP, and ESR values, although some may exhibit significantly elevated CRP and ESR levels. Importantly, there seems to be no discernible link between the number or location of bone lesions and these laboratory findings [35,53].

Imaging plays a crucial role in diagnosing CNO in pediatric patients. Whole-body MRI is commonly regarded as the preferred imaging modality and often considered the gold standard, although it may not be available in all centers. In most cases, patients typically undergo an initial radiographic assessment. Plain radiographs are easily accessible but lack sensitivity. Usual observations include osteolytic lesions surrounded by sclerosis and/or hyperostosis. However, MRI is the preferred choice due to its high sensitivity and the absence of radiation exposure [41]. Findings such as bone edema, sometimes accompanied by periosteal reaction and hyperostosis, as well as soft tissue inflammation, support the diagnosis of CNO [54,55]. The diagnostic breakdown of the 40 cases, with MRI utilized in 80% of cases to establish the diagnosis, underscores its predominant role in CNO diagnosis, highlighting its efficacy in identifying characteristic bone lesions associated with the condition. In some cases, a bone biopsy at multiple sites is performed in the initial stages of the diagnostic procedure, primarily to exclude infectious agents or malignant syndromes such as leukemia or intraosseous lymphoma. Common histologic findings include acute and/or chronic inflammation, bone marrow fibrosis, and osteonecrosis, while results may return normal [41].

Zhao et al. [54] conducted a survey among pediatric rheumatologists through the Childhood Arthritis and Rheumatology Research Alliance (CARRA) in 2016. The study aimed to determine physicians’ approaches to diagnosing CNO. X-ray (89%) emerged as the most commonly used diagnostic imaging modality, followed by regional MRI (78%) and bone scintigraphy (43%). The top three MRI findings considered indicative of active disease were bone edema (43%), periosteal reaction (37%), and soft tissue inflammation (28%). Vertebral compression, fracture, and physeal irregularity were identified as poor prognostic indicators. The Jansson [53] and Bristol [56] criteria, which incorporate the characteristic attributes of CNO, are the prevailing standards for assessment. The Bristol criteria [56] identify the best predictors for CNO. These criteria include a normal blood cell count; symmetric bone lesions; lesions with marginal sclerosis; normal body temperature; lesions located in the vertebrae, clavicle, or sternum; the presence of more than one radiologically confirmed lesion (OR 10.9); and a C-reactive protein (CRP) level equal to or greater than 1 mg/dL. A clinical scoring system for diagnosing CNO based on these predictors ranges from 0 to 63. A score of 39 or higher has a positive predictive value of 97% and a sensitivity of 68%. Jansson et al. [53] support a combination of specific criteria that must be met. The patient must exhibit characteristic clinical symptoms, including bone pain and localized swelling, without significant indications of local or systemic inflammation or infection. The radiological assessment should reveal distinct features on plain X-ray images, such as lytic areas, sclerosis, and new bone formation. Alternatively, using STIR MRI is preferable as it can depict bone marrow edema, potential bone expansion, lytic areas, and periosteal reactions. Additional diagnostic criteria include the involvement of more than one bone (or solely the clavicle) and CRP levels below a specific threshold (CRP ≤ 30 g/L). If the disease is unifocal, excluding the clavicle, or CRP levels exceed 30 g/L, the diagnosis can be confirmed through a bone biopsy. This biopsy should reveal signs of inflammation, such as the presence of plasma cells, osteoclasts, fibrosis, or sclerosis. Importantly, the biopsy should be performed without the patient being on antibiotic therapy, and bacterial growth should not be detected.

To manage pediatric IBD, well-established management strategies exist. For remission induction, systemic corticosteroids like prednisone are commonly used, while antibiotics such as metronidazole and ciprofloxacin are employed for perianal fistulizing disease. Additionally, oral 5-aminosalicylates (5-ASA) and anti-TNF-α treatment using biologics like infliximab and adalimumab play crucial roles. Immunomodulators like thiopurines and methotrexate serve as maintenance therapy [42]. In contrast, treatment strategies for CNO are primarily empirical due to the absence of published evidence from prospective trials. In many cases, patients have received antibiotic therapy before an official diagnosis of CNO is made. As the first-line treatment for CNO, NSAIDs are often utilized, with naproxen being the most chosen option [46,57]. NSAIDs can provide significant pain relief and reduce the number of bone lesions seen on MRI within as early as three months. In a recent study, Hedrich et al. documented a group of patients diagnosed with CNO/CRMO who underwent a one-year treatment regimen with naproxen. After this period, more than 50% of the patients experienced a symptom-free state. Nevertheless, it is important to note that only 27% of the patients achieved complete clinical remission without radiographic evidence of inflammation [58]. However, if children respond inadequately to NSAIDs after this period or if they continue to experience persistent pain and abnormal imaging findings, they are considered NSAID treatment failures, prompting the consideration of second-line treatments. In the absence of treatment guidelines, there is variation in the choice of medications for patients who do not respond to NSAIDs. Rheumatologists often differ in their selection and dosing of alternative medications. However, consensus treatment plans (CTPs) have been created based on the best available evidence and the practices of North American pediatric rheumatologists [59]. These CTPs are intended to provide guidance for the treatment of pediatric CNO cases that do not respond to NSAIDs or involve spinal issues. Utilizing these CTPs will help future studies determine the most effective treatments [41,60]. These secondary treatments include options such as methotrexate, anti-TNF-α, and bisphosphonates. The choice among these treatments may depend on the severity of the disease, with one or more medications used sequentially or concurrently after NSAID failure. While comparative effectiveness studies have not definitively determined the relative efficacy of these options, retrospective studies suggest that nonbiological DMARDs like methotrexate or sulfasalazine may have lower efficacy compared to anti-TNF-α and bisphosphonates. Anti-TNF-α, particularly in the form of monoclonal antibodies, appears to be more effective, especially when patients have additional conditions like inflammatory bowel disease (IBD) or enthesitis-related arthritis. Available data concerning the utilization of anti-TNF-α in the treatment of CNO is relatively scarce [59]. A limited-sized cohort study involving four participants, as documented by Eleftheriou et al., demonstrated a reduction in pain among children with CNO following treatment with infliximab (n = 3) and anakinra (n = 1) who subsequently switched to adalimumab [61]. Combining anti-TNF-α and bisphosphonates has shown promise in providing substantial disease control for CNO patients who do not respond well to NSAIDs. Simm et al. [62] and Miettunen et al. [63] showcased the efficacy of pamidronate in children diagnosed with NSAID-resistant CNO. In Simm’s study, over 80% of patients reported pain relief, while in Miettunen’s research, more than 90% of patients presented with resolved bone lesions on MRI after six months of pamidronate treatment.

Notably, infliximab emerged as an effective intervention in most cases showing significant responses, as combination therapy involving infliximab and azathioprine also demonstrated sustained remission for one year as described in the Van Ommen et al. case report [12], providing promising outcomes in managing both CNO and IBD. Corticosteroids played a pivotal role in ameliorating colitis and bone lesions associated with IBD; however, a noteworthy observation was the reappearance of symptoms following the cessation of corticosteroid therapy [15,19,20]. NSAIDs, including ibuprofen, naproxen and celecoxib, although controversial in their use for their gastrointestinal side effects, were in many cases one of the first treatment options for the CNO symptoms. The emergence of psoriasis has been identified as an adverse event associated with anti-TNF therapy, with specific instances reported in the literature. In a case presented by Dushnicky et al. [23], the use of adalimumab led to the development of psoriasis, prompting a therapeutic transition to ustekinumab, an interleukin-12/23 inhibitor. This case represents a distinctive approach in the management of both IBD and CNO utilizing ustekinumab. Additionally, Campbell et al. [14] reported a case where psoriasis manifested during the course of treatment with infliximab. The condition further exacerbated during subsequent treatments with adalimumab and tocilizumab. However, a notable improvement was observed following the discontinuation of adalimumab. This clinical scenario underscores the complexity of adverse events associated with anti-TNF-α therapy and the nuanced management required in such cases. Bousvaros et al. [4] describe a case where one year following the diagnosis, the patient experienced acute leukemia, leading to the necessity of bone marrow transplantation. Interestingly, at one year post-transplantation, there have been no reported symptoms of CNO or IBD.

The present manuscript is subject to certain limitations, primarily stemming from the review analysis and the subsequent retrospective design. The extensive time span of over 20 years (the oldest case report was published in 1996 whilst the most recent was published in 2022) during which the reported cases were documented introduces potential bias. Moreover, the data extracted from the literature may be incomplete, lacking longitudinal details, necessitating a cautious interpretation of each datum. Conversely, its retrospective nature and limited size (N = 40) preclude the derivation of generalizable results. Furthermore, the retrospective nature of the studies included in this review creates partially biased data. CNO is a clinical entity that has recently appeared in the literature and its diagnostic criteria in children remain obscure. Therefore, much of the case report data were retrospectively analyzed in the light of what has been proven nowadays about the disease. Lastly, the shared treatment pathways between CNO and IBD create difficulties in identifying the clinical remission of each disease after the initiation of treatment for one or the other clinical entity. Despite these constraints, it is noteworthy that this manuscript constitutes a comprehensive literature review. Future studies could enhance and expand upon the present findings in several ways. Implementing prospective study designs would provide a more rigorous approach, enabling researchers to collect data in real time and reducing retrospective biases. Longitudinal studies with an increased sample size could offer insights into the dynamic nature of the relationship between chronic CNO and IBD. Future studies should incorporate controlled comparisons, including both CNO patients with and without IBD, to better discern specific factors associated with the coexistence of these conditions. Comparisons could involve clinical characteristics, treatment outcomes, and disease progression. Undertaking long-term follow-up studies on patients with CNO and IBD could elucidate the course of these conditions over time. This approach would contribute valuable information on disease trajectories, treatment efficacy, and potential complications. Encouraging collaboration between various medical specialties, including rheumatology, gastroenterology, and immunology, would foster a holistic understanding of the interplay between CNO and IBD.

## 5. Conclusions

Chronic recurrent multifocal osteomyelitis (CRMO) should be taken into consideration when evaluating inflammatory bowel disease (IBD) patients experiencing unexplained bone pain or displaying abnormal areas of uptake on a bone scan. CRMO could potentially represent an uncommon extraintestinal manifestation of IBD, or it is possible that specific individuals have a genetic predisposition that makes them susceptible to both conditions [4]. The frequency of the CRMO diagnosis has increased significantly, a fact that is also confirmed by the literature. Therefore, vigilance for a timely diagnosis and initiation of treatment, as well as awareness of potential coexisting conditions such as IBD, are of great importance for the scientific community. This review serves as a critical foundation for further research and emphasizes the need for heightened clinical awareness of this co-occurrence by pediatricians, rheumatologists, gastroenterologists, as well as orthopedic surgeons.

## Figures and Tables

**Table 1:** Summary of case reports and case series identified by a systematic literature search.

First Author/Year	No. of Patients	Age	Sex	IBD Type	First Diagnosis	Time between 2 Clinical Presentations
Kotilainen (1996) [2]	1	29	F	CD	IBD	15 years
Omidi (1998) [3]	1	12	F	UC	CNO	
Bousvaros (1999) [4]	6	10	F	UC	CNO	5 years
		8	M	UC	CNO	5 years
		8	F	CD	CNO	3 months
		13	F	CD	CNO	6 months
		10	M	CD	CNO	3 years
		10	F	CD	CNO	18 months
Bazrafshan (2000) [5]	1	12	F	UC	IBD	3 years
Carpenter (2004) [6]	1	9	F	CD	CNO	19 months
Girschick (2007) [7]	1	9	F	CD	CNO	15 months
Bret (2008) [8]	1	29	M	CD	IBD	2 years
Morbach (2009) [9]	4	12	M	CD	CNO + IBD	Concomitantly
		15	M	CD	CNO + IBD	Concomitantly
		15	M	CD	CNO	6 years
		10	F	CD	CNO	8 months
Kim (2012) [10]	1	41	M	UC	IBD	8 months
Audu (2015) [11]	3	9	M	UC	CNO	1 month
		10	F	CD	CNO	2 years
		2	M	CD	CNO	
Christi van Ommen (2015) [12]	1	10	M	CD	CNO + IBD	Concomitantly
Ahmed (2018) [13]	1	11	F	UC	CNO	
Ramraj (2018) [14]	1	16	M	CD	IBD	Concomitantly
Campbell (2018) [15]	1 (of 5)	11	F	CD	IBD	6 months
Kołodziejczyk (2019) [16]	1	4,5	M	UC	CNO + IBD	Concomitantly
De Guerra (2019) [17]	1	15	F	UC	CNO	8 years
Lorenze (2020) [18]	1	12	F	UC	IBD	2 years
Fujisaki (2020) [19]	1	13	M	CD	IBD	Concomitantly
Kim (2021) [20]	1	21	M	UC	IBD	3 months
Ng (2021) [21]	1	12	F	UC	CNO	Concomitantly
Cantarelli (2021) [22]	1	10	M	CD	CNO	2 months
Dushnicky (2021) [23]	7	8	M	UC	IBD	5 years
		9	F	IBD-U	IBD	5 years
		13	F	CD	IBD	3 years
		9 months	M	CD	IBD	2.25 years
		9	F	UC	CNO + IBD	Concomitantly
		9	F	CD	IBD	1 year
		12	F	IBD-U	CNO	2 years
Goldfarb (2022) [24]	1	5	M	CD	CNO	Concomitantly
Mambelli (2022) [25]	1	10	F	UC	CNO	1 year

CD: Crohn’s disease; CNO: chronic nonbacterial osteomyelitis; F: female; M: male; IBD: inflammatory bowel disease; IBD-U: undifferentiated inflammatory bowel disease; UC: ulcerative colitis.

**Table 2:** Summary of case reports with different treatment modalities used for CNO and IBD.

First Author/Year	No. of Patients	Age	Sex	Treatment CNO	Treatment for IBD	Comorbidities	Response to Treatment—Outcome	Adverse Events—Complications
Kotilainen (1996) [2]	1	29	F	Prednisolone	PRED-metronidazole/5-ASA/AZA	SAPHO syndrome		Allergy to SSZ
Omidi (1998) [3]	1	12	F		Steroids	Pyoderma gangrenosum	One month after the cessation of corticosteroid therapy, a new hemorrhagic lesion appeared at the site of the scar-->new lesion of pyoderma gangrenosum.	
Bousvaros (1999) [4]	6	10	F	Steroids	Steroids/5-ASA/ 5-ASA enemas	Later developed sclerosing cholangitis	IBD treatment improved CNO symptoms.	
		8	M	Steroids/5-ASA	Steroids/5-ASA		Improved colitis and bone lesions, but flared with tapering.	Pancreatitis from 5-ASA, steroids
		8	F	Ibuprofen, naproxen	PRED/5-ASA	The first diagnosis was juvenile idiopathic arthritis instead of CNO		
		13	F		Steroids/AZA	Acute leukemia 1 year after diagnosis	One year after bone marrow transplantation, no symptoms of CNO/IBD.	
		10	M	Ibuprofen, indomethacin	Steroids/5-ASA			
		10	F	PRED	PRED/SSZ and SSZ enemas/MTX			
Bazrafshan (2000) [5]	1	12	F	Ibuprofen	PRED/5-ASA (MES, SSZ)/ pancolectomy		Pancolectomy before CNO diagnosis due to unresponsiveness to medical therapy.	
Carpenter (2004) [6]	1	9	F	NSAIDs	PRED/metronidazole/ 6-MP/AZA/INX		After 2 biopsies, CNO was diagnosed, although nonmultifocal, and an NSAID started. INX therapy was initiated at 400 mg per dose, with 3 doses given at 2-week intervals followed by monthly infusions for the following 6 months, and finally remission was maintained with 8-week intervals.	Herpes infection (stop metronidazole, 6-MP, weaning prednisone, start acyclovir). Steroid induced hyperglycemia. Insulin required.
Girschick (2007) [7]	1	9	F	Naproxen, PRED + SSZ	Budesonide/ 5-ASA/AZA	Fractures of thoracic vertebrae		Allergy to naproxen. Stomach pain with ibuprofen. Elevated liver enzymes from sulfasalazine.
Bret (2008) [8]	1	29	M	NSAIDs/APD/ INX	5-ASA/AZA/INX	Primary sclerosing cholangitis	NSAIDs provided little relief. Mesalazine (4 g/d for 1 year) followed by AZA (150 mg/d for 3 years) had no effect on the rheumatic manifestations. IV pamidronate (90 mg) failed to improve bone pain. INX therapy initiated at a dosage of 5 mg/kg; a remarkable response was noted after the second infusion.	NSAIDs caused gastrointestinal side effects.
Morbach (2009) [9]	4	12	M	N/A				
		15	M	N/A		CARD15: heterozygosity for the gene variants R702W and 1007fs in case 2 out of 4 checked		
		15	M	N/A				
		10	F	N/A				
Kim (2012) [10]	1	41	M	Intra-articular steroid injection/APD/MTX/steroids/NSAIDs (all together)	SSZ-MES/colectomy	Total colectomy/ileostomy	Improvement and resolution of symptoms after three weeks of treatment.	
Audu (2015) [11]	3	9	M	Hydrocortisone enemas, MES, steroids	Steroids/5-ASA		Seventeen months after presentation, symptoms resolved.	
		10	F		Steroids/APD	Eosinophilic esophagitis		
		2	M	IVIG/steroids (oral prednisolone)	Enteral feed/ AZA/gastrostomy	Splenic abscesses (partial splenectomy) Nonspecific rashes Non-necrotizing granulomatous proctitis Late IBD diagnosis	Treatment of non-necrotizing granulomatous proctitis treated with metronidazole-topical tacrolimus-prednisolone. Until 16 years old, dependent on enteral feeding.	
Christi van Ommen (2015) [12]	1	10	M	NSAIDs	INX/AZA/PRED/ modulen		Infliximab and azathioprine without relapses for one year.	
Ahmed (2018) [13]	1	11	F	Steroids	Steroids/5-ASA/AZA		CNO improved with 5-ASA, steroids, and immunomodulators.	
Ramraj (2018) [14]	1	16	M		Anti-TNF-α		One year after treatment initiation, complete resolution of bone lesions.	
Campbell (2018) [15]	1 (of 5)	11	F	NSAIDs/5-ASA/ APD/ADA	INX/MTX			Psoriasis induced by INX; worsening during treatment with ADA and tocilizumab; improvement after discontinuation of ADA.
Kołodziejczyk (2019) [16]	1	4,5	M	Ibuprofen/MTX/ADA	PRED/5-ASA/ AZA/MTX/ADA		Recurrence of symptoms after SSZ treatment. Recurrence after PRED termination. Clinical improvement with MTX and ADA.	
De Guerra (2019) [17]	1	15	F	Ibuprofen/steroids	PRED/SSZ/INX	Takayasu arteritis complicated with middle aortic syndrome Leg length discrepancy was surgically treated	Stable on INX 15 mg/kg every 4 weeks, MTX 15 mg/m^2^ weekly, and carvedilol for blood pressure.	
Lorenze (2020) [18]	1	12	F	IV methylprednisolone/PRED/MTX	INX		Complete resolution with steroids and MTX.	
Fujisaki (2020) [19]	1	13	M	INX	Prednisolone/ MES/elemental diet/INX		During steroid tapering, abdominal pain and diarrhea recurred, and heel pain developed.	
Kim (2021) [20]	1	21	M	NSAIDs	Prednisolone/ 5-ASA/AZA		Recurrence of abdominal pain and bloody diarrhea on day 12 of treatment, leading to discontinuation of NSAIDs. One month after discontinuation of prednisolone, left thigh pain recurred. Finally controlled with AZA.	
Ng (2021) [21]	1	12	F	Zoledronic acid	PRED/AZA/ ursodeoxycholic acid	Primary sclerosing cholangitis and auto-immune hepatitis	Initially treated with a single dose of intravenous zoledronic acid, which led to a significant clinical improvement and improved mobilization.	
Cantarelli (2021) [22]	1	10	M	ADA	ADA/INX + CDED+ MTX		ADA ineffective/remission was achieved after the application of CDED.	
Dushnicky (2021) [23]	7	8	M	Naproxen	5-ASA	Psoriasis	Use of NSAIDs was controversial but initiated.	
		9	F	Naproxen	Steroids	Latent TB-EBV hepatitis, Psoriasis vulgaris	Use of NSAIDs was controversial but initiated.	
		13	F	Celecoxib	Enteral nutritional therapy/MTX		Use of NSAIDs was controversial but initiated.	
		9 months	M	MTX	SSZ/ADA	Cow’s milk protein allergy	Use of NSAIDs was controversial, not added.	
		9	F	MTX/ADA	SSZ/ADA		Use of NSAIDs was controversial, not added.	
		9	F	INX/Ustekinumab	Enteral nutritional therapy/MTX/INX/ustekinumab	Pityriasis amiantacea Pityriasis alba Sebaceous nevus	Use of NSAIDs was controversial. Developed psoriasis, infliximab induced psoriasis, switched to ustekinumab (the first reported case of IBD and CNO treated with ustekinumab).	anti-TNF-α induced psoriasis
		12	F	NSAIDs/INX	5-ASA/SSZ/INX	Ehlers-Danlos syndrome/elevated fecal calprotectin, obtaineddue to physician awareness of the co-occurrence	Use of NSAIDs was controversial. Aminosalicylates/sulfasalazine were ineffective for IBD.	
Goldfarb (2022) [24]	1	5	M	Ketorolac	Parenteral nutrition/ IV pulsemethylprednisolone/INX	Hypercobalaminemia		
Mambelli (2022) [25]	1	10	F	Salazopyrin	Anti-TNF-α		Recurrence after 3 years of salazopyrin and initiation of anti-TNF-α.	

5-ASA: aminosalicylates; 6-MP: 6-mercaptopurine; ADA: adalimumab; APD: pamidronate disodium; AZA: azathioprine; anti-TNF-α: antitumor necrosis factor-α; CD: Crohn’s disease; CDED: Crohn’s disease exclusion diet; CNO: chronic nonbacterial osteomyelitis; EBV: Epstein–Barr virus; F: female; INX: infliximab; IV: intravenous; M: male; MES: mesalamine; MTX: methotrexate; NSAIDs: nonsteroidal anti-inflammatory drugs; IBD: inflammatory bowel disease; IBD-U: undifferentiated inflammatory bowel disease; IVIG: intravenous immunoglobulin; PRED: prednisone; SSZ: sulfasalazine; TB: tuberculosis; UC: ulcerative colitis.

**Table 3:** Patient characteristics.

Female	22/40 (55%)
Median age (range)	12 (9 months–41 years)
1st diagnosis CNO	21/40 (52.5%)
1st diagnosis IBD	14/40 (35%)
Crohn’s disease	23/40 (57.5%)
Ulcerative colitis	15/40 (37.5%)

CNO: chronic nonbacterial osteomyelitis; IBD: inflammatory bowel disease.

**Table 4:** Comorbidities.

Primary Sclerosing Cholangitis	3/40 (7.5%)
Psoriasis	2/40 (5%)
Pyoderma gangrenosum	1/40 (2.5%)
Takayasu arteritis	1/40 (2.5%)
Eosinophilic esophagitis	1/40 (2.5%)

## Data Availability

Data sharing is not applicable.
